# Synthesis, Spectroscopic, and Biological Studies of Mixed Ligand Complexes of Gemifloxacin and Glycine with Zn(II), Sn(II), and Ce(III)

**DOI:** 10.3390/molecules23051182

**Published:** 2018-05-15

**Authors:** Shimaa H. Sakr, Hazem S. Elshafie, Ippolito Camele, Sadeek A. Sadeek

**Affiliations:** 1 Department of Chemistry, Faculty of Science, Zagazig University, Zagazig 44519, Egypt; shimaa.chemist@yahoo.com (S.H.S.); s_sadeek@zu.edu.eg (S.A.S.); 2 School of Agricultural, Forestry, Food and Environmental Sciences, University of Basilicata, Viale dell’Ateneo Lucano, 85100 Potenza, Italy; ippolito.camele@unibas.it

**Keywords:** gemifloxacin, proton-nuclear magnetic resonance, fungicidal, antibacterial, antioxidant activity

## Abstract

Three novel mixed ligand metal complexes have been synthesized by the reaction of Zn(II), Sn(II), and Ce(III) with gemifloxacin (GMFX) in the presence of glycine (Gly) (1:1:1 molar ratio). The coordination possibility of the two ligands toward metal ions has been proposed in the light of elemental analysis, molar conductance, spectral infrared (IR), ultraviolet-visible (UV-Vis) and proton-nuclear magnetic resonance (^1^H NMR), and magnetic studies. Results suggest that GMFX and Gly interact with the metal ions as bidentate ligands. Electronic and magnetic data proposed the octahedral structure for all complexes under investigation. Antibacterial screening of the compounds was carried out in vitro against two Gram-positive bacteria, *Clavibacter michiganensis* and *Bacillus megaterium*, and two Gram-negative bacteria, *Escherichia coli* and *Xanthomonas campestris*. Antifungal activity was performed in vitro against *Rhizoctonia solani*, *Sclerotinia sclerotiorum*, *Aspergillus niger*, *Botrytis cinerea*, and *Penicillium digitatum*. The ligands and their complexes were also screened for their antioxidant activity. Results showed that some metal complexes showed more biological efficiency than the parent GMFX drug.

## 1. Introduction

**Scheme 1:**
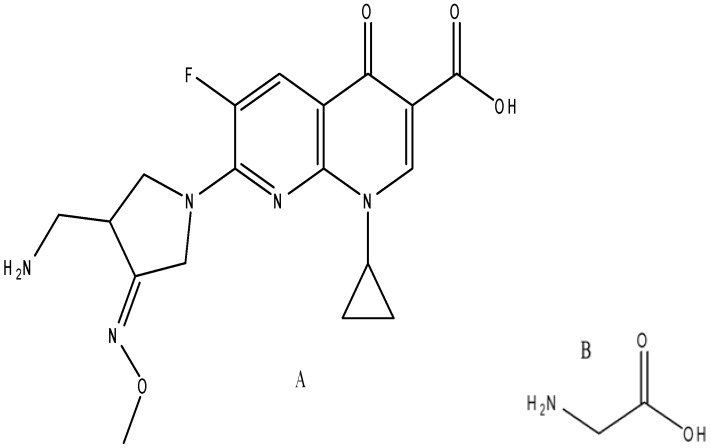
(**A**). 7-[(4*Z*)-3-(Aminomethyl)-4-methoxyimino-pyrrolidin-1-yl]-1-cyclopropyl-6-fluoro-4-oxo-1.8-naphthyridine-3-carboxylic acid (GMFX) and (**B**) amino acetic acid (Gly).

Quinolones are a group of synthetic antibacterial agents used in treatment of many infections [1,2,3]. Fluoroquinolone gemifloxacin (GMFX) (Scheme 1A) is a synthetic broad-spectrum antibacterial agent [4] used in the treatment of acute bacterial exacerbation of chronic bronchitis and mild-to-moderate pneumonia. GMFX has excellent in vitro activity against both Gram-positive (G+ve) and Gram-negative (G−ve) organisms, including potent antibacterial activity against respiratory tract infection pathogens [4,5]. GMFX acts by inhibiting DNA synthesis through the inhibition of both DNA gyrase and topoisomerase IV, which are essential for bacterial growth. Fluoroquinolone antibiotics have also been found to have anti-inflammatory and anticancer effects. Recently, some experiments were carried out and revealed that GMFX has an anticancer effect on colon cancer via inhibition of metastasis [6]. The interaction of metal ions with diverse deprotonated fluoroquinolones has been thoroughly studied [7,8]. In many cases, the metal complexes of drugs are more active than the parent compound [8,9].

From this point of view, diverse metal complexes with fluoroquinolones have been synthesized in an attempt to investigate the physicochemical properties and evaluate their biological properties [10,11] and potential antitumor activity in comparison with free fluoroquinolones [12,13,14]. Mixed ligand complexes play an important role in biological processes as exemplified by many instances in which enzymes are known to be activated by metal ions [11,12,13,14,15,16,17]. A good number of metal mixed-ligand complexes containing glycine (Gly) (Scheme 1B) with some other ligand are reported in the literature [18] where the Gly amino acid acts as bidentate ligand coordinating through the carboxylate oxygen and the amino nitrogen with octahedral geometry for metal chelates. The aim of the present work is to study the effect of changing atomic volume, atomic mass, and oxidation state of some metal ions (Zn(II), Sn(II), Ce(III)) with Cl^−^ as counter ion on the biological activity of gemifloxacin as antibacterial agent in the presence of Gly and to examine the mode of coordination and the biological properties of the resulting complexes. The synthesized complexes were characterized physicochemically by melting point, molar conductivity, magnetic property, elemental, infrared (IR), ultraviolet-visible (UV–Vis) and proton-nuclear magnetic resonance (^1^H NMR).

The biological characterization of GMFX and its synthesized metal complexes was carried out by an antifungal activity test activity against some phytopathogenic fungi such as; *Rhizoctonia solani* J.G. Kühn., *Sclerotinia sclerotiorum* (Lib.) de Bary., *Aspergillus niger* van Tieghem., *Botrytis cinerea* Pers., and *Penicillium digitatum* Sacc. On the other hand, the bactericidal effect of the above mentioned substances was performed against two G+ve bacteria *Xanthomonas campestris* Pammel and *Bacillus megaterium* de Bary and two G−ve bacteria *Escherichia coli* Migula and *Clavibacter michiganensis* Smith. In addition, antioxidant activity test has been also carried out for GMFX ligand and its synthesized metal complexes by using two different methods 2,2-diphenyl-1-picrylhydrazyl (DPPH) and 2,2′-azinobis (3-ethylbenzthiazoline-6-acid) (ABTS) for determine the radical scavenging activity (RSA%).

## 2. Results and Discussion

**Scheme 2:**
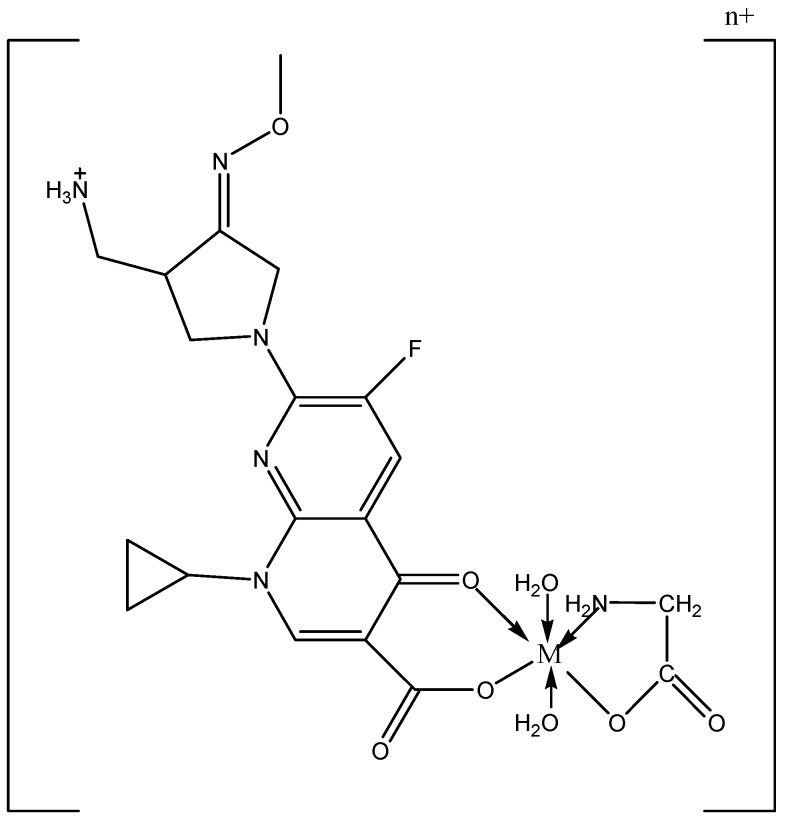
The coordination mode of Zn(II), Sn(II), and Ce(III) with mixed ligands. M = Zn(II), Sn(II), and Ce(III). *n* = 1 for Zn(II), Sn(II). *n* = 2 for Ce(III).

GMFX reacts with Zn(II), Sn(II), and Ce(III) in the presence of Gly as secondary ligand at 100 °C in ethanol to form solid complexes. The complexes were obtained as colored powdered materials. Table 1 summarizes the carbon, hydrogen, and nitrogen elemental analysis as well as melting point, magnetic measurements, and molar conductance. Moreover, the synthesized complexes were characterized using elemental analysis, IR, UV-Vis and ^1^H NMR spectra analyses. The molar ratio for all prepared complexes is GMFX:M:Gly = 1:1:1 which was established from the results of the chemical analyses and all the prepared complexes were air stable solids at room temperature. The IR spectroscopic data confirm the presence of water in the composition of the complexes [18] with the number of bound water molecules in these complexes being different. The structures of the complexes suggested from the elemental analysis agree quite well with their proposed formula. The metal ions are coordinated to a deprotonated carboxylate oxygen and pyridone oxygen of GMFX and Gly through the carboxylate oxygen and the amino nitrogen. Here, metal ions react with GMFX and Gly, forming complexes of monomeric structure where the metal ions are six coordinate and complete the coordination number with two water molecules (Scheme 2). The molar conductance values of the GMFX and their metal complexes in dimethyl formamide (DMF) with standard reference using 1 × 10^−3^ mol^−1^ solutions at room temperature were found to be in the range from 47.50 to 150.11 S cm^2^ mol^−1^. The data indicated that the complexes are electrolytes and the chloride ions were found as counter ions at all three complexes [19,20]. For our complexes, in order to verify that the chloride is ionic, their solutions were tested with aqueous solutions of AgNO_3_. A white precipitate was formed which indicated the presence of Cl^−^ as counter ions. The magnetic moments (as B. M.) of the complexes were measured at room temperature. The Zn(ΙΙ) and Sn(II) complexes were found to be of diamagnetic character with octahedral molecular geometry but the complex of Ce(ΙII) is susceptible and their magnetic moments were 2.34 μ_eff_.

The chloride content in the complexes was determined by using two methods; Mohr and Volhard. The lattice water for the three complexes was determined by heating an accurate weight of each complex from the ambient temperature up to ~120 °C, the weight loss for each chelate were calculated. The weight loss of Sn(II) and Ce(III) complexes are 2.74%, and 2.46%, respectively. These data support the proposed complexes’ chemical formulae and indicate the presence of one hydration water molecule for Sn(II) and Ce(III) complexes.

On the other hand, the measurement of the molar conductivity showed clear differences between the three prepared complexes (divalent and trivalent salt ions) as we have already specified in the manuscript (Table 1). Based on the above explanation, there is a steady relationship between increasing the conductivity of the compound and its solubility, as has been discussed by Uivarosi [21] who reported that the quinolone chelates of trivalent cations have shown an improved solubility compared to that of the free ligand and this behavior could be advantageous for pharmaceutical formulation.

### 2.1. Infrared Absorption Spectra

**Figure 1:**
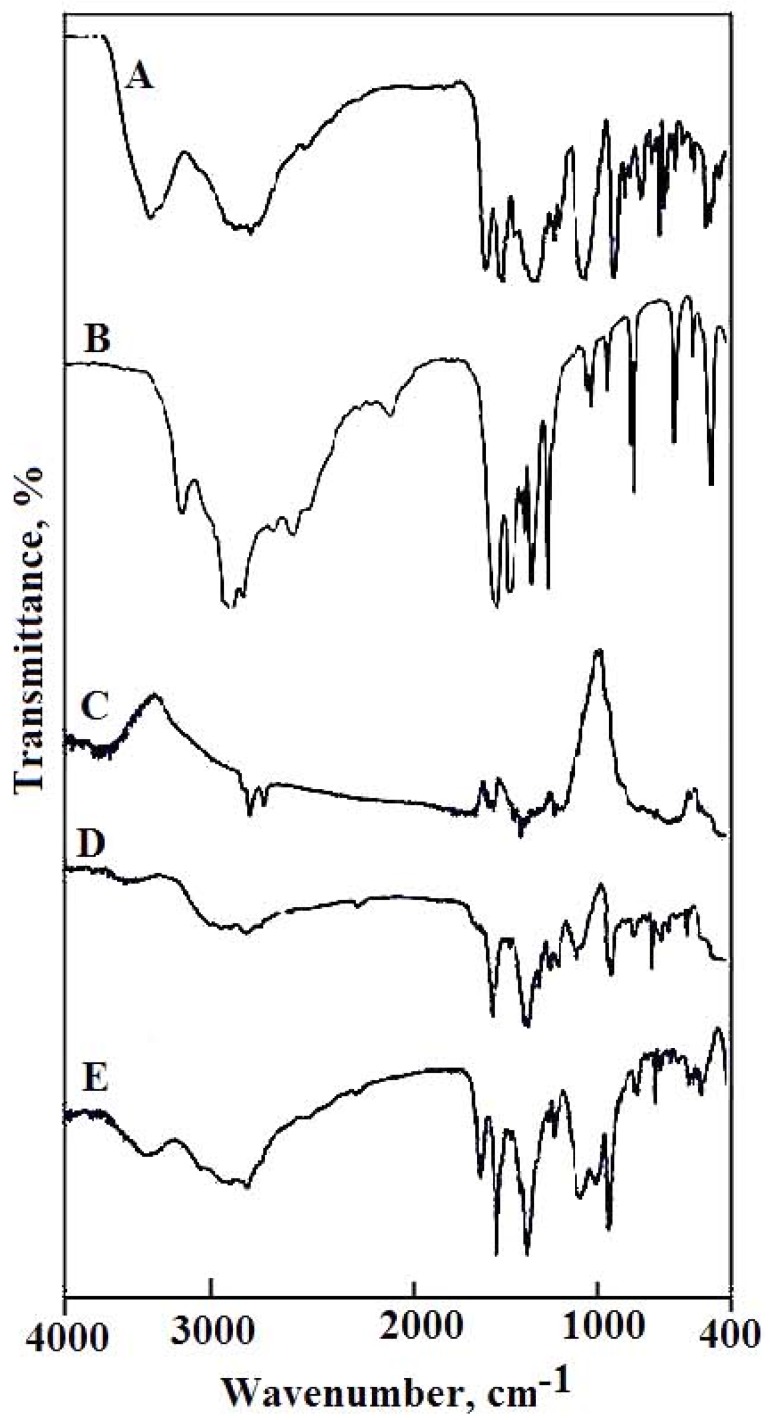
Infrared (IR) spectra for (**A**) gemifloxacin (GMFX), (**B**) glycine (Gly), (**C**) Sn(II), (**D**) Zn(II), and (**E**) Ce(III).

The infrared spectra of Zn(II), Sn(II), and Ce(III) complexes and free GMFX, Gly were measured as potassium bromide (KBr) discs. These spectra are shown in Figure 1 and the assignments are given in Table 2. The infrared spectra of the three complexes were compared with those of the free ligands in order to determine the site of coordination that may be involved in the chelation process. These peaks were expected to be involved in chelation. The position or the intensities of these peaks were expected to be changed upon complexation. The IR spectra of the complexes were very similar due to the same atoms of GMFX and Gly involved in the bonding to the metal. In most cases, the proposed mechanism of the interaction between GMFX and the metal cations was chelation between the metal ions through pyridone and carboxylic groups; therefore, it was important to give more attention to these group vibrations. The infrared spectra of the metal complexes exhibit a broad band between 3455 and 3411 cm^−1^ which corresponds to the ν(O–H) vibration and confirms the presence of water molecules in all complexes [18,22]. The two bands observed at 1720 and 1632 cm^−1^ in the spectrum of the free GMFX have been assigned to the stretching vibration of carboxylic ν(COOH) and the pyridone group ν(C=O), respectively [23], and at 1703 cm^−1^ for ν(C=O) carboxyl of Gly [24,25,26,27].

In the IR spectra of the complexes, the band corresponding to the carboxylic group appears with ∆ν = ν_as_(COO^−^) − ν_s_(COO^−^) > 200 cm^−1^, indicating carboxylic group involvement in monodentate coordination [28,29]. These changes in the IR spectra suggest that GMFX is bound to metal ions via the carboxylate oxygen atom [23]. Also, the second important band at 1634 cm^−1^, being attributed to the pyridone group, shifted to lower values at 1575 cm^−1^ for Sn(II), 1557 cm^−1^ for Zn(II), and 1559 cm^−1^ for Ce(III) being a good indication that this group is coordinated to the metal ions [30,31].

Regarding chelation through amino acids, the IR spectra exhibit significant features in the νNH_2_ and νCOO^−^ regions. It is worthwhile to mention here that free amino acids exist as zwitterions and the IR spectra of these cannot be compared entirely with those of metal complexes as amino acids in metal complexes do not exist as zwitterions. Free amino acids with NH_3_ functions in particular show νNH_3_ in the range of 3130–3030 cm^−1^. In the complexes, NH_3_ gets deprotonated and binds to metal through the neutral NH_2_ group. The transformation of NH_3_ to NH_2_ must result in an upward shift in the νNH_2_ of free amino acids. At the isoelectric point, they must show νNH_2_ in the region 3500–3300 cm^−1^ [26]. In the present complexes, the IR spectra showed characteristic bands in the region of 3459–3181 cm^−1^, which are lower compared to those of free νNH_2_. Hence, it can be concluded that the nitrogen of the amino group is involved in coordination. The IR spectra show strong evidence in support of the involvement of the carboxylate group in coordination.

In comparison with free amino acids, the νCOO^−^ (asymmetric) and νCOO^−^ (symmetric) record different shifts, which confirms the monodenticity [32] of the carboxylate group. The values of band shift ∆ν = ν_as_(COO^−^) − ν_s_(COO^−^) have indicated that the carboxylate group in Gly was chelated in a uni-negatively manner to the metal ions.

The spectra of the isolated solid complexes show a group of new bands with different intensities, being characteristic for ν(M–O) and ν(M–N). The ν(M–O) and ν(M–N) bands were observed at 689 and 601 cm^−1^ for Sn(II), at 692 and 642 cm^−1^ for Zn(II), at 693, 669, 640, 613, and 590 cm^−1^ for Ce(III), and being absent from the spectra of GMFX and Gly. This indicates coordination of GMFX and Gly through the pyridone, the carboxylate oxygen of GMFX, Gly, and the amino nitrogen of Gly.

### 2.2. Ultraviolet-Visible Spectroscopy Analysis

**Figure 2:**
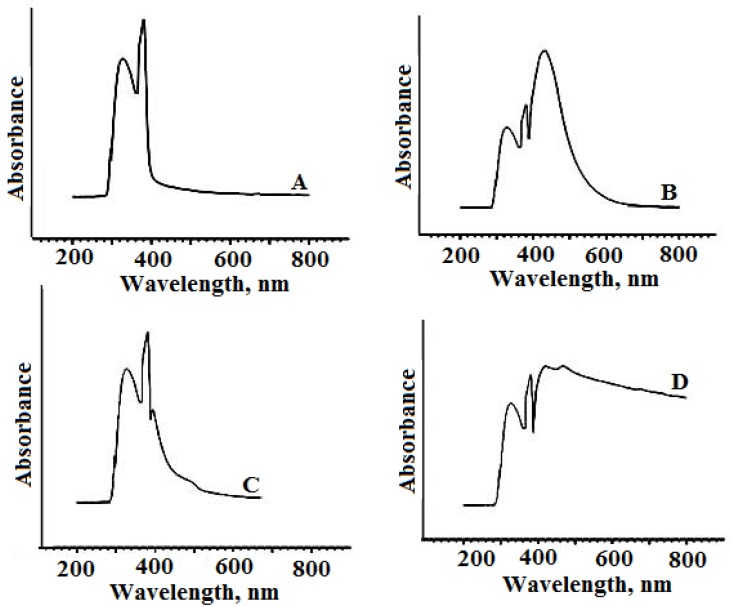
Ultraviolet-visible (UV-Vis). Spectrum for (**A**) GMFX, (**B**) Sn(II), (**C**) Zn(II), and (**D**) Ce(III).

Application of ultraviolet spectroscopy can be useful for structural determination of chelates since they all absorb in this region [24,33,34]. Formation of mixed ligand complexes was confirmed by the electronic absorption spectra. The electronic absorption spectra of the ligand along with the Zn(II), Sn(II), and Ce(III) complexes from 200 to 800 nm are shown in (Figure 2). Free GMFX showed bands at 213–245 nm and 276–343 nm which may be assigned to π–π* and *n*–π* transitions. Respectively. The shift of the absorption bands to higher or lower values and the appearance of new bands for the complexes are attributed to the mixed ligand complexation.

The decrease or increase of the absorption bands and the appearance of new bands upon coordination could be caused by the following effects: (1) the expected increase of the metal ion mass upon coordination; (2) the increase of the electron density on the metal ions by ligand, and (3) the decrease of the electron density on oxygen and nitrogen donor atoms. The complexes of Zn(II), Sn(II), and Ce(III) showed bands in the range of 402–468 nm, which may be assigned to ligand–metal charge transfer (Table 3).

### 2.3. Proton-Nuclear Magnetic Resonance Spectra Analysis

**Figure 3:**
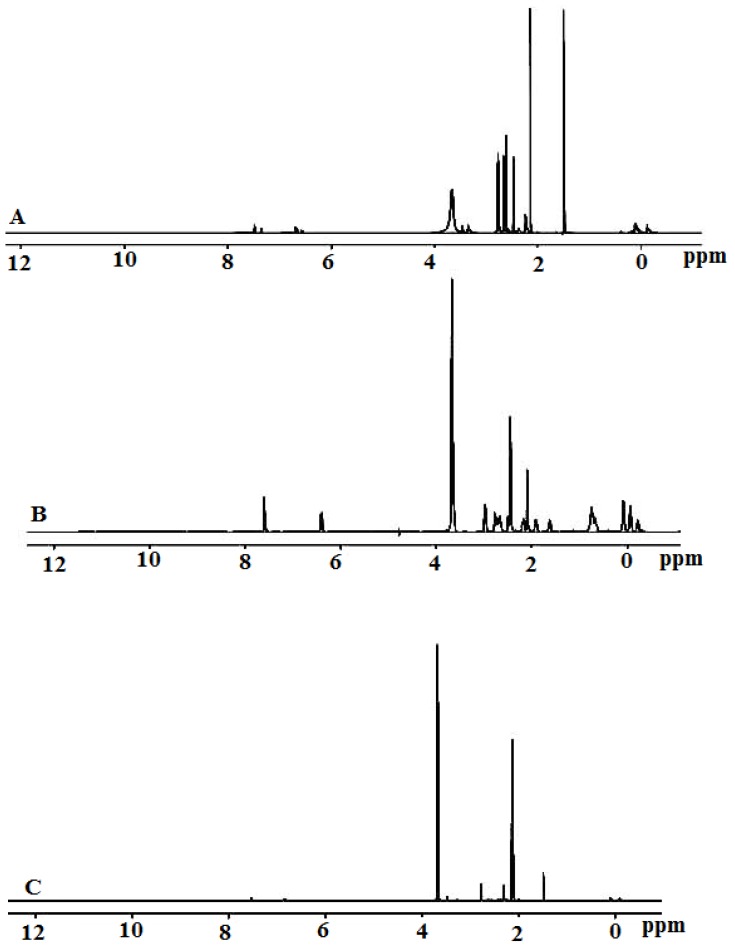
Proton-nuclear magnetic resonance (^1^H NMR) spectra for (**A**) [Sn(GMFX)(Gly)]Cl_2_, (**B**) [Zn(GMFX)(Gly)]Cl_2_·H_2_O, and (**C**) [Ce(GMFX)(Gly)]SO_4_·H_2_O.

The suggested molecular structures of the complexes were also proved by nuclear magnetic resonance (NMR) spectroscopy. The ^1^H NMR spectra of the ligands and their metal complexes in CD_3_OD displayed distinct signals with appropriate multiplets; the signal assignments are shown in (Figure 3). The ^1^H NMR spectrum obtained for GMFX shows peaks in the range δ = 8.02–8.72 ppm for the protons of an aromatic ring, a multiplet at δ = 1.14–1.33 ppm corresponding to –CH_2_ and –CH cyclopropane group, between 3.32 and 3.36 ppm for a –CH_2_ aliphatic group, and a singlet peak at δ = 11.0 ppm for the proton of carboxylic acid. The ^1^H NMR spectrum of glycine shows a peak at δ = 11.0 ppm for the proton of carboxylic acid and a peak at δ = 2.0 ppm for the amine (Table 4). 

Comparing the main signals of the complexes with those of GMFX and Gly, the ^1^H NMR spectra of the complexes showed chemical shift values that were only slightly changed compared with the free ligands, except for the carboxylic acid proton signal: the resonance of the carboxylic proton (COOH) was not detected in the spectra of the three complexes, suggesting coordination of GMFX and Gly through its carboxylation to oxygen atoms [24,25]. Also, the ^1^H NMR spectra for the complexes exhibit a new peak in the range of 4.50–4.89 ppm, due to the presence of water molecules in the complexes [35].

### 2.4. Biological Activities

#### 2.4.1. Antibacterial Activity

The results of antibacterial test showed that the tested ligands and its metal complexes were able to inhibit the growth of all studied bacterial strains in a dose dependent manner (Table 5). In particular, all GMFX–metal complexes demonstrated the highest significant activity against *E. coli* at 10,000 ppm compared to all other treatments. Whereas, GMFX 10,000 ppm and its Sn and Zn complexes at 1000 ppm showed moderate activity. In contrast. GMFX at 1000 and 100 ppm and its metals complexes at 100 ppm showed the lowest significant activity against *E. coli.*

In case of the antibacterial activity against *C. michiganensis*, the obtained results showed that GMFX and all its metal complexes inhibited its growth completely at all tested concentrations. In case of *X. campestris* and *B. megaterium*, the GMFX and all its metal complexes at 10,000 ppm showed the highest significant activity compared to all other treatments. The Ce(III)–GMFX complex at 100 ppm showed the lowest significant activity. The potential antibacterial efficacy of the tested ligand and its metal complexes is thought to depend on the specific toxicity of the studied metals. The low activity observed against *E. coli*, especially at low concentrations, could be due to the presence of lipopolysaccharides in the outer membrane of the G−ve bacteria, which make them inherently resistant to external agents. Cole et al. [36] have reported that the differences among G−ve and G+ve bacteria regarding the bactericidal effect are due to the structure of their cell walls, where the G−ve bacteria have a more complex cell wall composed of a thin peptidoglycan layer and an outer membrane containing lipopolysaccharides, whereas G+ve bacteria are protected by a wall predominantly composed by one type of macromolecule (pepidoglycan). The obtained results are in agreement with Sadeek et al. [37] who concluded that there is a significant increase in antibacterial activity of metal complexes compared to uncomplexed ligand.

#### 2.4.2. Antifungal Activity

Results of the in vitro antifungal activity testing of the studied ligand GMFX and its metal complexes against *R. solani* and *B. cinerea* showed that GMFX at 500 and 1000 ppm demonstrated the highest significant activity compared either to all other treatments or the two tested synthetic fungicides azoxystrobin and cycloheximide (Table 6). On the other hand, when the fungicidal activity against *S. sclerotiorum* was tested, the obtained results showed that azoxystrobin has significantly higher activity than GMFX. There were no significant differences between the Zn(II) and Ce(III) complexes. In the case of antifungal activity against *A. niger*, GMFX exhibited the highest significant activity at all tested concentrations and was similar to the Zn(II) complex and azoxystrobin. Regarding the activity against *P. digitatum*, GMFX at 1000 ppm and its Zn(II) complex had the highest significant activity, which was similar to both tested synthetic fungicides, and were followed by all other metal complexes.

The increased biological activity of metal chelates was explained by the concept of cell permeability and chelation theory. Upon chelation, the polarity of a metal ion is reduced due to the partial sharing of the positive charge with the donor groups of the ligand and as a consequence of overlap with the ligand orbitals. Chelation increases the delocalization of electrons over the whole chelate ring and thus increases the lipophilic nature of the central ion. This increase in lipophilicity enhances the passage of the complex through lipid membranes, facilitating the penetration of cells [38,39,40].

#### 2.4.3. Minimum Inhibitory Concentration of Fungicidal Activity

Minimum inhibitory concentration (MIC) tests were carried out for the most inhibited tested fungi (*B. cinerea* and *P. digitatum*). The effects of the different concentrations of GMFX and its metal complexes against the above-mentioned fungi were variable. In particular, MIC values in the case of *B. cinerea* were evaluated by 2000 ppm in case of the Ce(III)–GMFX complex and 2100 ppm in the case of the [Zn(II) or Sn(II)]–GMFX complexes and uncomplexed GMFX. Regarding *P. digitatum*, the MIC values were ordered in the following manner: Sn(II)–GMFX < Ce(III)–GMFX < free GMFX < Zn(II)–GMFX where the values were measured as 1400, 2100, 2200, and 2600 ppm, respectively. 

#### 2.4.4. Antioxidant Activity

**Figure 4:**
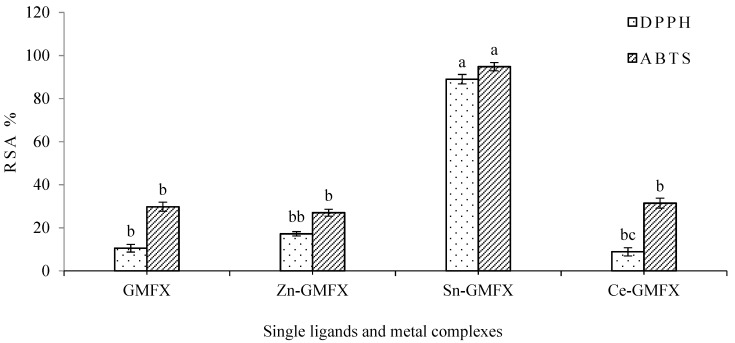
Radical scavenging activity (RSA,%) of GMFX and its metal complexes derived from 2,2-diphenyl-1-picrylhydrazyl (DPPH) and 2,2′-azinobis (3-ethylbenzthiazoline-6-acid) (ABTS) assays. Bars with different letters for each method indicate means values significantly different at *p* < 0.05 according to Tukey post hoc test. Data are expressed as the mean of three replicates ± SDs.

We have screened the antioxidant activity of GMFX and its metal complexes to evaluate their electron-donating activity. Results showed the highest significant antioxidant activity for the GMFX–Sn complex (*p* < 0.05) (Figure 4), compared to all other treatments. In particular, the DPPH and ABTS scavenging activity evidenced that Stannum presented the best percentage of antiradical activity. These results are in accordance with Bukhari et al. [41] and Chen et al. [42] who reported that the antioxidant activity of some ligands was increased by the presence of metal, such as Sn(IV). 

In any case, the antiradical activity of the GMFX–Sn complex could be due to its hydrogen donating ability, as reported by Corona-Bustamante et al. [43]. A possible exchange mechanism is that the proton from the GMFX ligand is donated and the charge is stabilized inside the entire complex, changing its charge delocalization capacity. The high scavenging activity of the Sn complex may explain its low antimicrobial activity, since the oxygenated compounds may contribute to the attachment to and destruction of the microorganism’s cell wall, hence increasing the permeability of cytoplasm fluid and finally leading to cell death.

## 3. Materials and Methods

### 3.1. Materials

All chemicals used for preparation of the complexes were of analytical reagent grade, being commercially available from different sources and used without further purification. Gemifloxacin was purchased from Obour Pharmaceutical Industrial Company (Cairo, Egypt). Glycine, AgNO_3_, K_2_CrO_4_, and ethanol (99.8%) were purchased from Fluka, Sigma Aldrich Company (Seelze, Germany). SnCl_2_·2H_2_O (98%) and ZnCl_2_ (98%) were purchased from Carlo Erba company (Milan, Italy). CeCl_3_·7H_2_O was purchased from Sigma Aldrich Company (Steinheim, Germany).

### 3.2. Synthesis of Mixed Ligand Metal Complexes

The faint brown solid complex [Sn(GMFX)(Gly)(H_2_O)_2_]Cl (Complex 1) was prepared by adding 0.5 mmol (0.112 g) stannous chloride SnCl_2_·2H_2_O in 50 mL ethanol drop-wise to a stirred suspended solution of 0.5 mmol (0.194 g) GMFX and 0.5 mmol (0.037 g) glycine(Gly) in 30 mL ethanol. All reactants were boiled in reflux apparatus in stirred conditions for about 5–6 h at 100 °C in water bath. Then, the prepared complex was subjected to the rotary evaporator for 30 min at 30 °C and 270 rpm until reach the completely dried complex. The faint yellow and beige solid complexes [Zn(GMFX)(Gly)(H_2_O)_2_]Cl·H_2_O (Complex 2) and [Ce(GMFX)(Gly)(H_2_O)_2_]Cl_2_·H_2_O (Complex 3) were prepared in a similar manner by using ethanol as a solvent and ZnCl_2_ and CeCl_3_, respectively, in 1:1:1 (GMFX:M:Gly) molar ratio with 0.5 mmol of ZnCl_2_, CeCl_3_·7H_2_O, GMFX, and glycine (Gly). We were not able to obtain appropriate monocrystals to perform X-ray diffraction analysis.

Complex (1)

(7-[(4*Z*)-3-(Aminomethyl)-4-methoxyimino-pyrrolidin-1-yl]-1-cyclopropyl-6-fluoro-4-oxo-1,8-naphthyridine-3-carboxylic acid)(aminoethanoic acid)(bihydrate) stannous chloride

Complex (2)

(7-[(*4*Z)-3-(Aminomethyl)-4-methoxyimino-pyrrolidin-1-yl]-1-cyclopropyl-6-fluoro-4-oxo-1,8-naphthyridine-3-carboxylic acid)(aminoethanoic acid)(bihydrate) zinc chloride monohydrate.

Complex (3)

(7-[(4*Z*)-3-(Aminomethyl)-4-methoxyimino-pyrrolidin-1-yl]-1-cyclopropyl-6-fluoro-4-oxo-1,8-naphthyridine-3-carboxylic acid) (aminoethanoic acid) (bihydrate)cerium bicholoride monohydrate.

### 3.3. Instruments

Elemental C, H, N analysis was carried out on a PerkinElmer CHN 2400 (Waltham, MA, USA). The percentage of metal ions was determined by using the atomic absorption method with a PYE-UNICAM SP 1900 spectrometer (Cambridge, UK) fit with the corresponding lamp. IR spectra were recorded from KBr discs using an FT/IR-460 Plus model Jasco-32 spectrophotometer (Easton, PA, USA) in the range from 4000 to 400 cm^−1^. ^1^H NMR spectra were recorded on a Varian 400 NMR spectrometer (Urbana, IL, USA) using CD_3_OD as solvent. Electronic spectra were obtained using T80 UV/Vis spectrometer (Taylors, SC, USA) PG Instruments Ltd. Magnetic susceptibilities of the powdered samples were measured on a Sherwood scientific magnetic balance (Cambridge, UK) using the Gouy method with Hg[Co(SCN)_4_] as calibrant. Molar conductance of 1 × 10^−3^ M solutions of ligand and metal complexes in DMF was measured at room temperature using a CONSORT K410 (Turnhout, Belgium). All measurements were carried out at ambient temperature with freshly prepared solutions. Melting points were recorded on a Buchi apparatus.

### 3.4. Antimicrobial Investigation

#### 3.4.1. Bactericidal Activity Test

The antibacterial test of the studied ligand and its metal complexes was investigated by the disc diffusion method of Bhunia et al. [20,44,45] against *X. campestris*, *B. megaterium, E. coli*, and *C. michiganensis* with some minor modifications. All strains were listed in National Collection of Plant Pathogenic Bacteria Catalogue (NCPPB) and have been cultivated, lyophilized, and freeze-conserving as pure cultures in the collection of the School of Agricultural, Forestry, Food and Environmental Sciences, University of Basilicata, Potenza, Italy. The bacterial suspension of each strain was prepared in sterile distilled water plus soft agar (0.7%) at concentration 10^8^ colony form unit (CFU) mL^−1^ (optical density, OD ≈ 0.2 nm). A mixture of 0.7% soft agar and bacterial suspension (9:1 *v*/*v*) was prepared and 4 mL of this suspension was poured into each 10 mL KB Petri dish 90 mm in diameter. Blank Discs (6 mm)-OXOID (Milan, Italy) were placed over KB-plate surfaces after complete solidification and 20 µL aliquots from each ligand and metal complexes at the following concentrations (1:5) 5000, 250, and 62.5 ppm.

#### 3.4.2. Fungicidal Activity Test

The antifungal screening was evaluated against five phytopathogenic fungi: *R. solani*, *S. sclerotiorum*, *A. niger*, *B. cinerea*, and *P. digitatum*. The tested fungi were previously identified by classical and molecular methods and stored in the mycotheca of School of Agricultural, Forestry, Food and Environmental Sciences, University of Basilicata, Potenza, Italy with the following collection numbers: *R. solani* 1312; *S. sclerotiorum* 593; *A. niger* 1008; *B. cinerea* 479, and *P. digitatum* 1395. Different concentrations of each treatment at 1000, 500, and 250 ppm was prepared in potato dextrose agar (PDA). Fourteen mL aliquots of previous prepared media supplemented with each treatment was poured into Petri dishes. After the complete dry off of the agar surface under laminar flow, 0.5 cm diameter of fungal disks square cut off from 96 h fresh cultures were singularly inoculated in the center of each Petri dish prepared as above. All plates were incubated at 24 ± 2 °C for 4 days under dark conditions. The diameter of fungal mycelium growth measured in mm. Petri dishes containing only PDA were also inoculated with fungal disks of the same fungi and used as a control. Each treatment was carried out in triplicate. The fungitoxicity was expressed as growth inhibition percentage (GIP) and calculated according to the formula of Zygadlo et al. [46] (Equation (1)), herein reported. Compared to synthetic fungicide azoxystrobin 1 µL mL^−1^ and cycloheximide 0.1 µg mL^−1^ to PDA nutrient medium:

GIP (%) = 100 × (GC − GT)/GC,(1)

where: GC = average diameter of fungus colony grown on PDA alone (control); GT = average diameter of fungus colony grown on PDA containing each treatment.

#### 3.4.3. Determination of Minimum Inhibitory Concentration (96-Well Microplate Method)

The MIC was considered as the fungicidal effect of each treatment, defined as the lowest concentration of each ligand and metal complexes that definitely inhibits the fungal growth. MIC was determined on 96-well culture plates by a micro-dilution method following the technical procedures of Lehtinen et al. [47]. Spore formation was ascertained after nine days incubation under a light microscope (Axioskop, ZEISS, West Germany). The stock solution of each treatment was prepared in Potato Dextrose Broth (PDB) at 1000, 1200, 1400, 1600, 1800, 2000, 2200, 2400, 2600, 2800, and 3000 µg mL^−1^. The absorbance of fungal growth was read at λ = 450 nm using the Elisa Microplate reader instrument (DAS s.r.l., Rome, Italy). All samples were tested in triplicate.

#### 3.4.4. Antioxidant Activity

In the present study, two tests were performed (ABTS and DPPH) following the methodological procedures of the basic principles of Martysiak-Żurowska and Wenta [48], as follows. In these trials, the above mentioned two assays were carried out to determine the radical scavenging activity (RSA%) of the studied ligand and its metal complexes at 100% utilizing the following formula:

Radical Scavenging Activity (RSA%) = (1 − A_t_/A_c_) × 100%;

where A_t_ is the absorbance of sample and A_c_ is the absorbance of colorimetric radical substance without sample.

##### DPPH: 2,2-diphenyl-1-picrylhydrazyl Assay

The DPPH method is considered one of the most useful techniques for measuring the antiradical activity of a compound and it depends on the use of the stable free radical DPPH. The electronic delocalization of this radical is responsible for its characteristic deep violet color and hence the compounds which will be able to donate a hydrogen atom will convert the DPPH radical into its reduced form (neutral stable molecule) and then the color switch from deep violet to pale yellow [49,50]. Stock radical solution (DPPH solution) was prepared by dissolving 20 mg of DPPH in 15 mL of ethanol. Thereafter, 1 mL of DPPH solution was diluted with 29 mL of ethanol.

Test procedures: 50 µL was diluted at (1:20) using 950 µL of DPPH solution and was incubated at darkness for 30 min at room temperature. Later on, all samples were centrifuged at 8000 rpm for 5 min and the absorbance was measured at 515 nm in triplicate using a UV-Vis spectrophotometer (LKB Biochrom 4050 Ultrospec II, Arizona, USA) considering the value of the reference sample (ethanol). The stock solution of DPPH was prepared fresh and all determinations were carried out in triplicate.

##### ABTS: 2,2′-azinobis (3-ethylbenzthiazoline-6-acid) Assay

The ABTS method is considered the most sensitive method and is characterized by higher repeatability for observing the kinetics of specific enzymes. The detectability and sensitivity of ABTS are higher than the DPPH assay. 

Stock radical solution. ABTS solution was prepared by dissolving 38 mg of ABTS in 10 mL of a aqueous sodium persulphate solution (2.45 mM). and the solution was conserved in darkness for 16 h at room temperature. Then, 1 mL of stock ABTS^•+^ solution was diluted with 29 mL of ethanol.

Test procedures: 20 µL of each studied ligand and the metal complexes was diluted at (1:50) using 980 µL of radical ABTS^•+^ solution and was incubated after that in darkness at room temperature for 2 h. All samples were centrifuged at 8000 rpm for 5 min and the absorbance was measured at 734 nm by using UV-Vis spectrophotometer (LKB Biochrom 4050 Ultrospec II) considering the value of reference sample (ethanol). The stock solution of ABTS was prepared fresh and all determinations were carried out in triplicate.

### 3.5. Statistical Analysis

The outcomes of the biological tests were statistically analyzed using statistical package for the social sciences (SPSS) (version 13.0. Prentice Hall, Chicago, IL, USA, 2004). Experimental data were expressed as mean ± SD and comparisons were employed by Tukey post-hoc test for detecting any significance differences among different treatments at *p* < 0.05.

## Figures and Tables

**Table 1:** Elemental analysis and physico-analytical data for gemifloxacin (GMFX), glycine (Gly), and its metal complexes.

Compounds M.wt. and M.F.	Yield%	Mp/°C	Color	Found (Calcd.) (%)					μ_eff_ (B. M.)	Λ S cm^2^ mol^−1^
				C	H	N	M	Cl		
**GMFX 389.4 (C_18_H_20_FN_5_O_4_)**	-	220	White brown	55.50 (55.52)	5.18 (5.17)	17.98 (17.99)	-	-	-	47.50
**Gly 75.07 (C_2_H_5_NO_2_)**	-	233	White	31.98 (32.00)	6.64 (6.66)	18.63 (18.66)	-	-	-	50.77
**(1) 619.89 (ZnC_20_H_31_N_6_O_9_FCl)**	78	310	Faint yellow	38.69 (38.71)	4.97 (5.00)	13.49 (13.55)	10.50 (10.55)	5.72 (5.72)	-	97.58
**(2) 655.20 (SnC_20_H_29_N_6_O_8_FCl)**	82	330	Faint brown	36.60 (36.63)	4.39 (4.42)	12.79 (12.82)	17.98 (18.11)	5.38 (5.41)	-	86.34
**(3) 730.10 (CeC_20_H_31_N_6_O_9_FCl_2_)**	95	340	Beige	32.82 (32.87)	4.18 (4.24)	11.42 (11.50)	19.12 (19.19)	9.70 (9.72)	2.34	150.11

Where: M.wt.: molecular weight; M.F.: molecular formula; Mp: melting point; Calcd.: calculated values; μ_eff_.: effective magnetic moment; S: siemens (Ohm^−1^).

**Table 2:** Infrared frequencies (cm^−1^) and tentative assignments for (**A**) GMFX, (**B**) Gly, (**C**) Sn(II), (**D**) Zn(II), and (**E**) Ce(III).

A	B	C	D	E	Assignments
3533w	3522w 3478w	3341mbr	3545w	3375mbr	ν(O–H); H_2_O; COOH
3156w	3133vw	3067vw	3044sh	3089vw	ν(N–H)
3044w		3009m		3022w	
2943s	2955m	2889w	2974m	2955m	ν(C–H); aliphatic
2867vw	2843m	2766w	2871vw	2822vw	
2778m	2767w	2711vw	2800m	2756w	
2712ms	2722w		2758w	2716m	
	2712m				
1732vs	1703vs	-	-	-	ν(C=O); COOH
-	1620vs	1620vs	1622m	1624s	ν_as_(COO^−^)
1624vs	1589s	1574s	1574s	1570vs	ν(C=N)
	1539m				
1466vs	1494s	1485vs	1462vs	1485s	–CH; deformations of CH_2_
	1474s				
1385m	1377s	1393s	1385vs	1393s	ν_s_(COO^−^)
1367sh		1378w	1350sh		
1331ms	1322m	1304ms	1300m	1308s	δ_b_(–CH_2_)
1261vs	1285s	1269s	1269s	1265s	ν(C–O), ν(C–N) and ν(C–C)
1223w	1211sh	1189vw	1184m	1184m	
1188m	1172vw	1144w	1146m	1146m	
1156m	1146ms				
1089m	1034s	1103m	1038ms	1107m	δ_r_(–CH_2_)
1034s		1072sh		1034s	
		1038m			
989vw	989w	989vw	945ms	945s	–CH-bend; phenyl
968m	937ms	941ms	899m	895m	
933m	894w	911vw	844m	829m	
887ms	867m	829m	822w		
833ms	833ms		800vw		
822vw					
795m	783m	779m	778w	778m	δ_b_(COO^−^)
767vw	721s	745s	752m	748s	
748m		710w	711m	710m	
710m					
656w	644vw	625ms	633ms	667vw	ν(M–O). ν(M–N) and ring deformation
622m	621ms	578m	583m	629m	
571ms	559s	544w	544w	578w	
550vw	494ms	502m	505s	544m	
486m	436m	478w	478sh	502m	
478vw	406m	433w	444vw	478sh	
436s		413w	422w	440w	

Keys: s = strong, w = weak, v = very, m = medium, br = broad, sh = shoulder, ν = stretching, δb = bending.

**Table 3:** UV-Vis spectral data of the free ligand GMFX and its metal complexes.

Assignments (nm)	GMFX	GMFX Metal Complexes		
		Zn(II)	Sn(II)	Ce(III)
π–π* transitions	326	332	330	332
*n*–π* transitions	380	390	388	392
**Ligand-metal charge transfer**	-	402	432	420, 468

**Table 4:** ^1^H NMR values (ppm) and tentative assignments for GMFX, Gly ligands and its metal complexes.

GMFX	Gly	Zn(II)	Sn(II)	Ce(III)	Assignments
1.14–1.33	-	1.29–1.32	1.20–1.33	1.14–1.33	δH.–CH_2_ and –CH cyclopropane
2.71	2.00	2.71	2.72	2.71	δH.–^+^NH_2_
3.32–3.36	3.59 -	3.32–3.84 4.59	3.36–3.99 4.89	3.21–3.99 4.50	δH.–CH_2_ aliphatic δH. H_2_O
8.02–8.72	-	8.40–8.71	8.40–8.71	8.40–8.71	δH.–CH aromatic
11	11	-	-	-	δH.–COOH

**Table 5:** Antibacterial activity of the studied GMFX ligand and its metal complexes compared to four synthetic antibiotics.

Bacterial Growth Inhibition %				
	G−ve		G+ve	
Treatments µg/mL	*E. coli*	*X. campestris*	*C. michiganensis*	*B. megaterium*
GMFX 10,000	68.9 ± 0.1b	75.6 ± 0.1ab	90.0 ± 0.9ab	54.4 ± 0.2bc
GMFX 1000	61.1 ± 0.2bc	57.8 ± 0.3bc	86.0 ± 0.4b	43.3 ± 0.1c
GMFX 100	55.5 ± 0.6c	50.0 ± 0.2c	85.0 ± 0.6b	37.8 ± 0.1cd
Sn–GMFX 10,000	82.2 ± 0.3a	82.2 ± 0.2a	97.0 ± 0.7a	56.7 ± 0.1bc
Sn–GMFX 1000	65.6 ± 0.2b	62.2 ± 0.2bc	86.0 ± 0.5b	44.4 ± 0.1c
Sn–GMFX 100	52.2 ± 0.1c	52.2 ± 0.1c	84.0 ± 0.6b	38.9 ± 0.2cd
Zn–GMFX 10,000	80.0 ± 0.1a	83.3 ± 0.2a	95.0 ± 0.8a	57.8 ± 0.2bc
Zn–GMFX 1000	65.6 ± 0.1b	60.0 ± 0.2bc	89.0 ± 0.6ab	48.9 ± 0.2c
Zn–GMFX 100	52.0 ± 0.4c	51.1 ± 0.2c	85.0 ± 0.4b	37.8 ± 0.3cd
Ce–GMFX 10,000	83.0 ± 0.3a	72.2 ± 0.2b	98.0 ± 0.6a	61.1 ± 0.2b
Ce–GMFX 1000	60.0 ± 0.2bc	60.0 ± 0.3bc	91.0 ± 0.0ab	41.1 ± 0.1c
Ce–GMFX 100	50.0 ± 0.2c	42.2 ± 0.1d	92.0 ± 0.0ab	30.0 ± 0.1d
Ampicillin 100 µg/mL	80.0 ± 0.3a	74.4 ± 0.3ab	98.0 ± 0.5a	32.2 ± 0.1cd
Streptomycin 50 µg/mL	51.1 ± 0.1c	55.6 ± 0.1bc	96.0 ± 0.7a	53.3 ± 0.1bc
Kanamycin 50 µg/mL	68.9 ± 0.2b	71.1 ± 0.2b	98.0 ± 0.4a	57.8 ± 0.1bc
Cephaloxacin 30 µg/mL	53.3 ± 0.2c	60.0 ± 0.2bc	93.0 ± 0.9a	84.0 ± 0.14a
Control KB	0.0 ± 0.0d	0.0 ± 0.0e	0.0 ± 0.0c	0.00 ± 0.0e

Where: GMFX: gemifloxacin; Sn: Tin; Zn: Zinc; Ce: Cerium; KB: King’s B media. Values are recorded as the mean of bacterial inhibition percentage (%) from three replicates ± SDs. Values followed by the different letter in each vertical column for each bacterium are significantly different according to Tukey post hoc test at *p* < 0.05.

**Table 6:** Antifungal activity of GMFX and its metal complexes.

		Fungal Mycelium Growth Inhibition %				
	Conc. µg/mL	*R. solani*	*S. sclerotiorum*	*A. niger*	*B. cinerea*	*P. digitatum*
GMFX	1000	78.1 ± 0.22a	71.9 ± 0.04ab	69.0 ± 0.13a	91.9 ± 0.13a	95.3 ± 0.11a
	500	66.3 ± 0.18b	34.4 ± 0.67b	63.4 ± 0.13ab	86.3 ± 0.09ab	83.1 ± 0.13b
	250	50.0 ± 0.0c	0.0 ± 0.0d	45.0 ± 0.36c	60.6 ± 0.13b	76.3 ± 0.09b
Sn–GMFX	1000	28.1 ± 0.22d	6.3 ± 0.45cd	56.9 ± 0.09b	51.3 ± 0.09c	76.3 ± 0.09b
	500	6.3 ± 0.0e	0.0 ± 0.0d	53.1 ± 0.09bc	45.6 ± 0.13cd	69.4 ± 0.13bc
	250	0.0 ± 0.0f	0.0 ± 0.0d	48.4 ± 0.22bc	37.5 ± 0.0d	63.1 ± 0.04bc
Zn–GMFX	1000	15.6 ± 0.22de	26.9 ± 0.31b	65.6 ± 0.09ab	51.3 ± 0.09c	90.6 ± 0.13a
	500	7.5 ± 0.09e	18.8 ± 1.34bc	64.4 ± 0.09ab	45.0 ± 0.09cd	75.0 ± 0.18b
	250	0.0 ± 0.0f	11.3 ± 0.18c	58.8 ± 0.36b	37.5 ± 0.0d	64.4 ± 0.13bc
Ce–GMFX	1000	43.1 ± 0.3cd1	21.9 ± 0.67b	64.4 ± 0.09ab	50.0 ± 0.0c	71.3 ± 0.09b
	500	18.1 ± 0.13d	12.5 ± 0.89c	63.8 ± 0.18ab	37.5 ± 0.0d	65.6 ± 0.13bc
	250	3.1 ± 0.22e	6.3 ± 0.45cd	41.6 ± 0.58c	37.5 ± 0.0d	61.3 ± 0.09c
Azoxystrobin 1 µL/mL		26.9 ± 0.13d	26.9 ± 0.13d	80.0 ± 0.09a	72.2 ± 0.13a	58.1 ± 0.13b
Cycloheximide 0.1 µL/mL		14.4 ± 0.13de	14.4 ± 0.13de	10.6 ± 0.31c	49.7 ± 0.31bc	46.9 ± 0.22cd
PDA ctrl		0.00 ± 0.0f	0.00 ± 0.0f	0.0 ± 0.0d	0.0 ± 0.0cd	0.0 ± 0.0d

Where: GMFX: gemifloxacin; Sn: Tin; Zn: Zinc; Ce: Cerium; PDA: Potato Dextrose Agar. Values are recorded as the mean of fungal mycelium growth inhibition (%) from three replicates ± SDs. Values followed by the different letter in each vertical column for each fungus are significantly different according to Tukey post hoc test at *p* < 0.05.

## References

[1] Psomas G., Tarushi A., Efthimiadou E.K. (2008). Synthesis, characterization and DNA-binding of the mononuclear dioxouranium(VI) complex with ciprofloxacin. Polyhedron.

[2] Lesher G.Y., Froelich E.J., Gruett M.D., Bailey J.H., Brundage R.P. (1962). 1,8-Naphthyridine derivatives: A new class of chemotherapeutic agents. J. Med. Pharm. Chem..

[3] Nelson J.M., Chiller T.M., Powers J.H., Angulo F.J. (2007). Fluoroquinolone-resistant Campylobacter species and the withdrawal of fluoroquinolones from use in poultry: A public health success story. Clin. Infect. Dis..

[4] Turel I. (2002). The interactions of metal ions with quinolone antibacterial agents. Coord. Chem. Rev..

[5] Andriole V.T. (2000). The Quinolones.

[6] Kuhlmann J., Schaefer H.G., Beermann D., Kuhlmann J., Dalhoff A., Zeile A.J. (1998). Quinolone Antibacterials.

[7] Johnson D.M., Jones R.N., Erwin M.E. (1999). Anti-streptococcal activity of SB-265805 (LB20304), a novel fluoronaphthyridone, compared with five other compounds, including quality control guidelines. Diagn. Microbiol. Infect. Dis..

[8] Grossman R., Rotschafer J., Tan J. (2005). Antimicrobial treatment of lower respiratory tract infections in the hospital setting. Am. J. Med..

[9] Kan Y.K., Hsu Y.L., Chen Y.H., Chen T.C., Wang J.Y., Kuo P.L. (2013). Gemifloxacin, a fluoroquinolone antimicrobial drug, inhibits migration and invasion of human colon cancer cells. BioMed Res. Int..

[10] Patel R.N., Singh N., Shukla K.K., Gundla V.L.N., Chauhan U.K. (2006). Synthesis, characterization and biological activity of ternary copper(II) complexes containing polypyridyl ligands. Spectrochim. Acta Part A.

[11] Viossat B., Daran J., Savouret G., Morgant G., Greenaway F.T., Dung N., Pham-Tran V.A., Sorenson J.R.J. (2003). Low-temperature (180 K) crystal structure, electron paramagnetic resonance spectroscopy, and propitious anticonvulsant activities of CuII2(aspirinate)4(DMF)2 and other CuII2(aspirinate)4 chelates. J. Inorg. Biochem..

[12] Dimiza F., Papadopoulos A.N., Tangoulis V., Psycharis V., Raptopoulou C.P., Kessissoglou D.P., Psomas G. (2010). Biological evaluation of non-steroidal anti-inflammatory drugs-cobalt(II) complexes. Dalton Trans..

[13] Turel I., Golobic A., Klavzar A., Pihlar B., Buglyo P., Tolis E., Rehder D., Sepcic K. (2003). Interactions of oxovanadium(IV) and the quinolone family member—Ciprofloxacin. J. Inorg. Biochem..

[14] Lopez-Gresa M.P., Ortiz R., Perello L., Latorre J., Liu-Gonzalez M., Garcia-Granda S., Perez-Priede M., Canton E. (2002). Interactions of metal ions with two quinolone antimicrobial agents (cinoxacin and ciprofloxacin). Spectroscopic and X-ray structural characterization. Antibacterial studies. J. Inorg. Biochem..

[15] Efthimiadou E.K., Katsarou M.E., Karaliota A., Psomas G. (2008). Copper(II) complexes with sparfloxacin and nitrogen-donor heterocyclic ligands: Structure-activity relationship. J. Inorg. Biochem..

[16] Efthimiadou E.K., Thomadaki H., Sanakis Y., Raptopoulou C.P., Katsaros N., Scorilas A., Karaliota A., Psomas G. (2007). Structure and biological properties of the copper(II) complex with the quinolone antibacterial drug N-propyl-norfloxacin and 2,2′-bipyridine. J. Inorg. Biochem..

[17] Katsarou M.E., Efthimiadou E.K., Psomas G., Karaliota A., Vourloumis D. (2008). Novel copper(II) complex of N-propyl-norfloxacin and 1,10-phenanthroline with enhanced antileukemic and DNA nuclease activities. J. Med. Chem..

[18] Mohamed G.G., Abd El-Halim H.F., El-Dessouky M.M.I., Mahmoud W.H. (2011). Synthesis and characterization of mixed ligand complexes of lomefloxacin drug and glycine with transition metals. Antibacterial, antifungal and cytotoxicity studies. J. Mol. Struct..

[19] Geary W.J. (1971). The use of conductivity measurements in organic solvents for the characterisation of coordination compounds. Coord. Chem. Rev..

[20] Abu-Dief A.M., Nassr L.A.E. (2015). Tailoring, physicochemical characterization, antibacterial and DNA binding mode studies of Cu(II) Schiff bases amino acid bioactive agents incorporating 5-bromo-2-hydroxybenzaldehyde. J. Iran. Chem. Soc..

[21] Uivarosi V. (2013). Metal Complexes of Quinolone Antibiotics and Their Applications: An Update. Molecules.

[22] Bhunia A.K., Johnson M.C., Ray B. (1988). Purification, characterization and microbial spectrum of a bacteriocin produced byPediococcus acidilactici. J. Appl. Bacteriol..

[23] Nakamoto L. (1963). Infrared spectra of inorganic and coordination compounds. J. Chem. Educ..

[24] Sadeek S.A., EL-Shwiniy W.H., El-Attar M.S. (2011). Synthesis, characterization and antimicrobial investigation of some moxifloxacin metal complexes. Spectrochim. Acta A Mol. Biomol. Spectrosc..

[25] Sadeek S.A., EL-Shwiniy W.H. (2010). Metal complexes of the fourth generation quinolone antimicrobial drug gatifloxacin: Synthesis, structure and biological evaluation. J. Mol. Struct..

[26] Macias B., Martinez M., Sanchez A., Dominguez A. (1994). A physico-chemical study of the interaction of ciprofloxacin and ofloxacin with polivalent cations. Int. J. Pharm..

[27] Turel I., Bukovec N., Farkas E. (1996). Complex formation between some metals and a quinolone family member (ciprofloxacin). Polyhedron.

[28] Abdel-Rahman L.H., Abu-Dief A.M., Adam M.S., Hamdan S.K. (2016). Some New Nano-sized Mononuclear Cu(II) Schiff Base Complexes: Design, Characterization, Molecular Modeling and Catalytic Potentials in Benzyl Alcohol Oxidation. Catal. Lett..

[29] Abdel-Rahman L.H., Abu-Dief A.M., Moustafa H., Hamdan S.K. (2017). Ni(II) and Cu(II) complexes with ONNO asymmetric tetradentate Schiff base ligand: Synthesis, spectroscopic characterization, theoretical calculations, DNA interaction and antimicrobial studies. Appl. Organometal. Chem..

[30] Petrenko V.I., Radysh H.V. (2013). Treatment of drug resistant destructive pulmonary tuberculosis: Gemifloxacin and other fluoroquinolones clinical efficiency and tolerance at the end of initial phase of treatment. Likar. Sprav..

[31] Kasselouri S., Hadjiliadis N. (1990). Interaction of *cis*-Pd(guo)_2_Cl_2_ with amino acids. Inorg. Chim. Acta.

[32] Bellamy L.J. (1975). The Infrared Spectra of Complex Molecules.

[33] Abdel-Rahman L.H., El-Khatib R.M., Nassr L.A.E., Abu-Dief A.M., Lashin F.E. (2013). Design, characterization, teratogenicity testing, antibacterial, antifungal and DNA interaction of few high spin Fe(II) Schiff base amino acid complexes. Spectrochim. Acta Part A.

[34] Abdel-Rahman L.H., Abu-Dief A.M., Aboelez M.O., Abdel-Mawgoud A.H. (2017). DNA interaction, antimicrobial, anticancer activities and molecular docking study of some new VO(II), Cr(III), Mn(II) and Ni(II) mononuclear chelates encompassing quaridentate imine ligand. J. Photochem. Photobiol. B.

[35] Macias B., Villa M.V., Rubio I., Castineiras A., Borras J. (2001). Complexes of Ni(II) and Cu(II) with ofloxacin Crystal structure of a new Cu(II) ofloxacin complex. J. Inorg. Biochem..

[36] Cole A., Goodfield J., Williams D.R., Midley J.M. (1984). The complexation of transition series metal ions by nalidixic acid. Inorg. Chim. Acta.

[37] Sadeek A.S., Abd El-Hamid S.M., El-Aasser M.M. (2015). Synthesis, characterization, antimicrobial and cytotoxicity studies of some transition metal complexes with gemifloxacin. Monatsh Chem..

[38] Tumer M., Koksal H., Sener M.K., Serin S. (1999). Antimicrobial activity studies of the binuclear metal complexes derived from tridentate Schiff base ligands. Transit. Met. Chem..

[39] Imran M., Iqbal J., Iqbal S., Ijaz N. (2007). In Vitro Antibacterial studies of ciprofloxacin-imines and their complexes with Cu(II), Ni(II), Co(II), and Zn(II). Turk. J. Biol..

[40] Patel N.H., Parekh H.M., Patel M.N. (2007). Synthesis, physicochemical characteristics, and biocidal activity of some transition metal mixed-ligand complexes with bidentate (NO and NN) Schiff bases. J. Pharm. Chem..

[41] Bukhari S.B., Memon S., Mahroof-Tahir M., Bhanger M.I. (2009). Synthesis, characterization and antioxidant activity copper–quercetin complex. Acta Part A Mol. Biomol. Spec..

[42] Chen W., Sun S., Cao W., Liang Y., Song J. (2009). Antioxidant property of quercetin-Cr(III) complex: The role of Cr(III) ion. J. Mol. Struct..

[43] Corona-Bustamante A., Viveros-Paredes J.M., Flores-Parra A., Peraza-Campos A.L., Martínez-Martínez F.J., Sumaya-Martínez M.T., Ramos-Organillo Á. (2010). Antioxidant activity of Butyl- and Phenylstannoxanes derivedfrom 2-, 3- and 4-Pyridinecarboxylic acids. Molecules.

[44] Abdel-Rahman L.H., El-Khatib R.M., Nassr L.A.E., Abu-Dief A.M., Ismael M., Seleem A.A. (2014). Metal based pharmacologically active agents: Synthesis, structural characterization, molecular modeling, CT-DNA binding studies and in vitro antimicrobial screening of iron(II) bromosalicylidene amino acid chelates. Spectrochim. Acta Part A Mol. Biomol. Spectrosc..

[45] Abdel-Rahman L.H., Abu-Dief A.M., El-Khatib R.M., Abdel-Fatah S.M. (2016). Sonochemical synthesis, DNA binding, antimicrobial evaluation and in vitro anticancer activity of three new nano-sized Cu(II), Co(II) and Ni(II) chelates based on tri-dentate NOO imine ligands as precursors for metal oxides. J. Photochem. Photobiol. B.

[46] Zygadlo J.A., Guzman C.A., Grosso N.R. (1994). Antifungal properties of the leaf oils of *Tagetes minuta* L. and *Tagetes filifolia* Lag. J. Essent. Oil Res..

[47] Lehtinen J., Järvinen S., Virta M., Lilius E.M. (2006). Real-time monitoring of antimicrobial activity with the multiparameter microplate assay. J. Microbiol. Meth..

[48] Martysiak-Żurowska D., Wenta W. (2012). Comparison of ABTS and DPPH methods for assessing the total antioxidant capacity of human milk. Acta Sci. Pol. Technol. Aliment..

[49] Bafna A., Mishra S. (2005). Actividad antioxidante in vitro del extracto de metanol de los rizomas de Curculigo orchioides Gaertn. ARS Pharm..

[50] Abdel-Rahman L.H., Abu-Dief A.M., Newair E.F., Hamdan S.K. (2016). Some new nano-sized Cr(III), Fe(II), Co(II), and Ni(II) complexes incorporating 2-((*E*)-(pyridine-2-ylimino)methyl)napthalen-1-ol ligand: Structural characterization, electrochemical, antioxidant, antimicrobial, antiviral assessment and DNA interaction. J. Photochem. Photobiol. B Biol..

